# Impact of a qualitative assessment approach for neonatal abstinence syndrome management: experience of a European reference center

**DOI:** 10.1186/s13052-024-01788-6

**Published:** 2024-10-29

**Authors:** Mariana Cortez Ferreira, Ana Moura Figueiredo, Joaquim Pitorra, Joana Mesquita da Silva

**Affiliations:** 1 Neonatology Department, Maternidade Bissaya Barreto, Centro Hospitalar e Universitário de Coimbra, Coimbra, Portugal; 2 Obstetrics Department, Maternidade Bissaya Barreto, Centro Hospitalar e Universitário de Coimbra, Coimbra, Portugal

**Keywords:** Neonatal abstinence syndromes, Neonatal substance withdrawal, Neonates, Substance related disorder

## Abstract

**Background:**

The management of infants at risk of neonatal abstinence syndrome (NAS) remains challenging. In 2000 Maternidade Bissaya Barreto implemented a strategy based on the qualitative assessment of neonates and in 2018 the Eat, Sleep, Console (ESC) approach, a tool based on similar concepts, was created. The aim is to assess the efficacy of a qualitative assessment of infants at risk, compare it with the ESC approach and report temporal trends of NAS in a European hospital.

**Methods:**

Retrospective cohort study of all infants of mothers with a history of drug abuse during pregnancy admitted to a tertiary European centre between January 2010 and December 2021. The therapeutical decision was guided by a qualitative assessment of the newborn’s well-being. The ESC approach was retrospectively determined. Pharmacologic treatment was used as a last resort. The clinical outcomes and therapeutic strategies employed were evaluated. Statistical association was evaluated. The incidence rate per 1000 births was calculated and temporal trend differences were identified.

**Results:**

A total of 79 neonates at risk were included, of whom 40 (50.6%) developed NAS. Consolability was the most affected criterion (35.0%), followed by feeding difficulties (12.5%). Sleep was affected less frequently (5.0%). Overall, 37.5% of infants failed to meet at least one of the criteria. All neonates with a positive ESC failed the qualitative assessment (*p* = 1.000) After optimization of nonpharmacologic measures, drug therapy was still necessary in four cases (10.0% of infants with the syndrome). The incidence rate of NAS decreased from 3.9 per 1000 births in 2010 to 0.0 per 1000 births in 2021 (*p* = 0.025).

**Conclusion:**

The qualitative assessment of the infant based on the ability to feed, sleep and be consoled correctly identified neonates at risk and led to a significant reduction in the use of drug therapy. The incidence rate of NAS decreased during the study period.

## Introduction

Neonatal abstinence syndrome (NAS) results from the sudden discontinuation of chronic fetal exposure to substances (typically opioids) taken by the mother during the prenatal period [1–3]. This syndrome has a significant impact on the health of affected infants, with longer hospital stays, admission to the Neonatal Intensive Care Unit (NICU) and increased morbidity and mortality rates [2, 4]. Additionally, opioid exposure has been linked to intrauterine growth restriction, low birth weight and small head circumference [5–7], as well as placental dysfunction and impaired brain development [8].

A dramatic increase in the incidence of NAS has been observed in the United States over recent decades [9–14], with an estimated annual incidence rate of 20 per 1000 births in 2016 [9]. It is estimated that 55–94% of infants exposed to opioids *in utero* develop NAS [15], and different studies report a need for pharmacologic treatment in 17–77% of cases [1, 11, 16] and median hospital stays of 14,5 to 16,1 days [17–21]. In contrast, available data regarding NAS in Europe is lacking and given the differences in opioid usage and public health systems, it is not possible to extrapolate the findings to the European context.

Clinically, NAS is characterized by non-specific signs and symptoms indicative of central nervous system irritability, autonomic overreactivity, and gastrointestinal tract dysfunction These manifestations are thought to reflect the sites where the opioid receptors are concentrated [2, 10, 16]. The Finnegan Neonatal Abstinence Score (FNAS) created by Finnegan et al. in 1975 [22], is the most used diagnostic and assessment tool for infants with NAS [1, 2, 10, 11, 23–25]. Nevertheless, many authors have highlighted several shortcomings including a lack of clinical validation [1, 11, 24, 25], complex implementation [1], the necessity for direct contact with the neonate [11, 24] and the use of subjective criteria with uncertain clinical relevance [1, 11, 24, 25]. Consequently, the need for an alternative system that is easier to implement in routine clinical practice has remained.

The Maternidade Bissaya Barreto (MBB) is a tertiary Maternal and Child Hospital reference centre with vast experience in the care and treatment of addicted pregnant women and their children. A multidisciplinary team is responsible for the follow-up of every case from the prenatal period to the third year of life. After three years of age, all children are referred to a Neurodevelopmental Pediatrician at the local Pediatrics Hospital. All pregnant women with a history of drug abuse are followed in the Early Intervention Unit (EIU) where they are frequently evaluated and undergo regular screenings for active drug abuse, evaluation of comorbidities, screening for fetal disease and, if indicated, prescription and monitoring of opioid replacement therapy. The monitoring by social workers is constant. After birth, rooming-in is arranged and breastfeeding is encouraged. At discharge, all newborns are referred to the EIU where they will have regular follow-up appointments where safety, growth and neurodevelopment are closely monitored.

Similarly to other authors’ experience with FNAS [1, 11, 24, 25], MBB encountered challenges when utilising this instrument. The primary impediments to the utilisation of this tool were identified as interobserver variability and the time-consuming nature of its implementation. Consequently, in 2000 MBB implemented an alternative strategy to guide the therapeutic management of newborns with NAS. Since then, neonates have undergone a qualitative assessment of their well-being. This assessment considered the ability to feed, appropriate weight gain according to standard care, the ability to sleep for a duration exceeding one hour after feeding, and the ease of consolation as the primary factors in guiding therapeutic decision-making. If one or more of these elements was found to be compromised, intervention for NAS was indicated.

In 2018 the Eat, Sleep, Console (ESC) approach was created by Grossman et al. [11] and applies similar concepts to MBB [9, 12, 24–26]. This method has gained increasing attention [9, 12, 24–26] and evaluates three key abilities of the neonate – eat, sleep and be consoled. NAS was considered controlled by the authors if the baby was able to breastfeed effectively (or take at least 30 ml from a bottle per feed), sleep at least 1 h undisturbed and, if crying, to be consoled within 10 min. In contrast to the qualitative approach previously described, this method employs strict cut-offs. If the newborn does not meet one of these criteria, nonpharmacologic interventions are maximized. In the event that these interventions prove ineffective, pharmacologic treatment is started. Nevertheless, despite the initial enthusiasm surrounding this method, currently there is some conflicting data. Whereas some studies showed shorter lengths of stay and initiation of pharmacologic treatment [27–29], there were no differences regarding long-term safety outcomes [28] and one study failed to show any benefit [30].

The aim of this study was to assess the results of a qualitative assessment for therapeutic guidance of NAS. As a secondary objective, we determined if the subset of neonates at risk identified through this method was similar to the ones that would have been identified if the ESC approach had been used instead. Furthermore, we also report temporal trends of NAS in a European hospital.

## Methods

### Study design and case selection

Quantitative, retrospective cohort study of all consecutive infants born of mothers with drug use admitted to MBB from January 2010 to December 2021. Inclusion criteria were: (1) infants with ≥ 35 weeks gestation and (2) maternal history of prenatal opioid use/maternal toxicology screen positive. Exclusion criteria included (1) infants with major birth defects; (2) hypoxic ischemic encephalopathy, metabolic disorder, stroke, intracranial hemorrhage or meningitis diagnosed by 48 h of life; (3) major surgical interventions by 48 h of life. In accordance with the institution’s protocol, all infants were managed in the newborn nursery with their mother, except in the event that admission to the NICU was required due to the presence of additional comorbidities. In the absence of an absolute contraindication (such as active substance abuse or maternal human immunodeficiency virus infection), breastfeeding was always encouraged. The use of opioid substitution treatment during pregnancy or after birth was not considered a contraindication to breastfeeding.

Non-pharmacological interventions were the primary method of management for all neonates, employed from birth and throughout the entire hospital stay. These measures included a rooming in a low-stimulation environment (characterised by low light and noise levels, whispering, relaxing music and nature sounds), minimal manipulation, regular skin-to-skin contact and gentle rocking, swaddling, use of pacifier, non-nutritive sucking and on-demand feeding.

The therapeutical decision was guided by the newborn’s well-being qualitative assessment method used at MBB: ability to feed along with appropriate weight gain according to standard of care, the ability to sleep longer than an hour after feeding and the ease of consolation (qualitative). All neonates at risk were evaluated every two to six hours by a neonatal care nurse specialist and by a certified neonatologist at least twice daily (or more frequently if considered necessary). In the event that NAS was deemed to be unresponsive to the initial intervention (failure to meet one of the criteria), a second evaluation by a certified neonatologist was performed. If failure to meet the criteria persisted, the nonpharmacologic interventions were optimized. Pharmacologic treatment with methadone was considered a last resort when all other measures had failed.

A retrospective assessment with the ESC approach was conducted in order to identify neonates considered at risk with this method and determine if they were equally identified by the qualitative assessment.

### Data collection

Clinical data were obtained by reviewing all perinatal and neonatal medical records. Long-term follow-up information was obtained from EIU medical records up to three years of age and from Neurodevelopmental follow-up appointments if older.

Diagnosis of NAS was based on the presence of clinical manifestations and maternal history of drug abuse during pregnancy. The following signs and symptoms were considered clinical manifestations of NAS: irritability, high-pitched crying, decreased sleep, tremors, increased muscle tone, hyperactive deep tendon reflexes/hyperactive Moro reflex, seizures, feeding difficulties, vomiting, uncoordinated and constant sucking, diarrhea, excessive weight lost, increased sweating, fever, flushing/mottling, nasal stuffiness, frequent yawning and sneezing, and tachypnea [2, 10, 16]. The highest FNAS score was recorded. Long-term neurodevelopmental outcomes were obtained. Neuropsychomotor developmental delay was considered when the global development quotient was equal to or smaller than 70 in the Griffiths Scales of Child Development [31]. Specific developmental disability was determined by a certified speech therapist. Cerebral palsy was diagnosed according to the definition of the European Cerebral Palsy Network [32]. Autism spectrum disorder was diagnosed by a positive Autism Diagnostic Observation Schedule, Second edition (ADOS-2) [33]. Attention deficit hyperactivity disorder diagnostic was clinical and based on the Diagnostic and Statistical Manual, Fifth edition (DSM-5) criteria for the disease [34].

Maternal sociodemographic characteristics (age, parity, number and type of drugs abused, opioid substitution therapy, alcohol and tobacco use, chronic medication, gestational diabetes, TORCH – toxoplasmosis, others (syphilis, hepatitis B), rubella, cytomegalovirus, and herpes simplex – and hepatitis C infections) were retrieved. Additionally, labor (type of delivery, gestational age, birthweight and 5-minute Apgar score) as well as neonatal data (sex, diagnosis of NAS and age at diagnosis, length of stay, NICU admission, pharmacologic treatment, type of feeding, maximum weight loss, comorbidities and mortality) were included in the analysis.

For the purpose of this study, this information was used to conduct a retrospective evaluation of the ESC approach. Failure to meet any criteria (eating, sleeping or consolability) was registered.

### Data analysis

The incidence rate per 1000 births was calculated by dividing the number of new cases per the total number of births in a given year and multiplying by 1000 [35].

Statistical analysis was performed using IBM^®^SPSS^®^ Statistics version 26. Categorical variables are presented as frequencies and percentages, and continuous variables as means and standard deviations (SD) if normally distributed or as medians and interquartile range (IQR) if non-normally distributed. Normal distribution was verified through the Kolmogorov-Smirnov test or skewness and kurtosis (maximum tolerated interval of -1 to 1). Two groups were considered: with NAS and without NAS. Bivariate analysis to determine association was performed using the χ2 test (or Fisher exact test if expected cases in a cell < 5) for categorical variables and independent samples t-test (if normally distributed) or Mann-Whitney U test (if non-normally distributed) for continuous variables. A Mann-Kendall test was used to determine if there was a significant variation in the number of cases during the study period.

All reported p values are two-tailed with values inferior to 0.05 indicating statistical significance. P-values shown underwent Bonferroni correction to compensate for multiple comparisons.

Approval was obtained from the local Ethics Committee (process number OBS.SF.203/2021).

## Results

A total of 79 neonates were included. Median gestational age was 38 weeks. Preterm labor occurred in 11 cases (13.9%). The remaining baseline characteristics are presented in Table 1.

**Table 1:** Characteristics of overall sample and comparison between groups with and without NAS

Maternal characteristics	Total (*n* = 79)	With NAS (*n* = 40)	Without NAS (*n* = 39)	*p*-value^#^
Maternal age – mean ± SD [years]	31.4 ± 5.1	31.3 ± 5.2	31.6 ± 5.1	0.753
Primipara - n (%)	24 (30.4)	14 (34.1)	10 (26.3)	0.366
Drugs - n (%)				**0.019**
One	19 (24.1)	5 (14.7)	14 (40.0)	
Two or more	50 (63.3)	29 (85.3)	21 (60.0)	
Drugs - n (%)*				
Heroin	60 (75.9)	35 (97.2)	25 (69.4)	**0.006**
Cocaine	49 (62.0)	26 (65.0)	21 (53.8)	0.426
Cannabinoids	21 (26.6)	8 (23.5)	13 (37.1)	0.657
Substitution therapy - n (%)				
None	10 (2.7)	1 (2.5)	9 (23.1)	**0.021**
Methadone	53 (67.1)	32 (80.0)	21 (53.8)	**0.039**
Buprenorphine	16 (20.2)	7 (17.5)	9 (23.1)	1.000
Other abuses - n (%)				
Tobacco	58 (73.4)	30 (96.8)	28 (82.4)	0.107
Alcohol	3 (3.8)	0 (0.0)	3 (11.5)	0.236
Chronic medication - n (%)*				
Psychiatric				
Benzodiazepine	20 (25.3)	15 (39.5)	5 (12.8)	0.064
Antidepressant	18 (22.8)	10 (26.3)	8 (20.5)	1.000
Antipsychotic	9 (11.4)	5 (13.2)	4 (10.3)	1.000
Other				
Antiretroviral	5 (6.3)	3 (7.9)	0 (0.0)	1.000
Anticonvulsant	3 (3.8)	0 (0.0)	1 (2.6)	1.000
Antihistamine	2 (2.5)	3 (7.9)	2 (5.1)	1.000
Anticholinergic	1 (1.3)	1 (2.6)	0 (0.0)	0.952
Corticoids	1 (1.3)	1 (2.6)	0 (0.0)	0.952
Gestational diabetes - n (%)	6 (7.6)	2 (5.0)	4 (10.3)	0.432
TORCH infection - n (%)	39 (49.4)	23 (57.5)	16 (41.0)	0.143
HCV infection - n (%)	38 (48.1)	23 (57.5)	15 (38.5)	0.090
Education level - n (%)				0.443
Lower Secondary	17 (21.5)	9 (45.0)	8 (53.3)	
Upper Secondary	16 (20.2)	9 (45.0)	7 (46.7)	
Tertiary	2 (2.5)	2 (10.0)	0 (0.0)	
**Perinatal characteristics**				
Cesarean delivery - n (%)	22 (27.8)	11 (27.5)	11 (28.2)	0.944
Gestational age - median (IQR) [weeks]	38.3 (35–41)	38.2 (35–41)	38.3 (35–41)	0.911
Birthweight - mean ± SD [gram]	2704 ± 486	2649 ± 456	2761 ± 514	0.306
Male - n (%)	41 (51.9)	20 (48.8)	21 (55.3)	0.732
5-minute Apgar score < 7 - n (%)	0 (0.0)	0 (0.0)	0 (0.0)	-
**Neonatal characteristics**				
Length of stay - median (IQR) [days]	7.0 (2–36)	8.5 (4–33)	6.0 (2–36)	**0.029**
NICU admission - n (%)	28 (35.4)	20 (50.0)	31 (79.5)	**0.006**
NAS as main cause	1 (1.3)	1 (5.0)	0 (0.0)	
Length of stay at NICU - median (IQR) [days]	7.5 (1–33)	7.0 (1–31)	8.5 (1–33)	0.582
Breastfeeding - n (%)	62 (78.5)	31 (77.5)	31 (79.5)	0.830
Exclusive	33 (53.2)	12 (30.0)	21 (53.8)	**0.032**
Maximum weight loss from birthweight - mean ± SD [%]	8.3 ± 3.6	9.0 ± 3.2	7.5 ± 4.0	0.062
Comorbidities - n (%)	13 (16.5)	7 (17.9)	6 (15.4)	0.761
**Long-term outcomes**				
Follow-up time - median (IQR)	8.0 (1–12)	9.0 (1–12)	7.0 (1–12)	**0.029**
Neurodevelopmental outcomes - n (%)				
Neuropsychomotor developmental delay	11 (13.9)	5 (12.5)	6 (15.4)	1.000
Specific learning disability	1 (1.3)	1 (2.5)	0 (0.0)	1.000
Cerebral palsy	1 (1.3)	0 (0.0)	1 (2.6)	1.000
Autism spectrum disorder	2 (2.6)	1 (2.5)	1 (2.6)	1.000
Attention deficit hyperactivity disorder	9 (11.4)	6 (15.0)	3 (7.7)	1.000

*More than one option may be present in each case; ^#^With Bonferroni correction; HCV, hepatitis C; IQR, interquartile range; NICU, Neonatal Intensive Care Unit; SD, standard deviation; TORCH, toxoplasmosis, others (syphilis, hepatitis B), rubella, cytomegalovirus, and herpes simplex

Neonatal abstinence syndrome was diagnosed in 40 cases (50.6%), with a median age at diagnosis of 2 days. NAS was more frequent in infants born to mothers with a history of multiple drug abuse (*p* = 0.019) and those with a history of heroin use (*p* = 0.006). The use of methadone during pregnancy as a substitution therapy was more common in the NAS group (*p* = 0.039). No differences were found regarding maternal education level.

The median length of stay was 8.5 days *versus* 6.0 days in those without NAS (*p* = 0.029). Over a third of the infants (*n* = 28) were admitted to the NICU. The primary reason for admission was social risk (*n* = 10; 35.7%), followed by hyperbilirubinemia requiring intensive phototherapy (*n* = 6; 21.4%) and transient tachypnea of the newborn (*n* = 5; 35.7%). Only one infant was admitted due to neonatal seizures caused by NAS.

Failure to pass the qualitative assessment was observed in 15 infants (37.5%). These same 15 infants were the only ones to have a positive ESC score (*p* = 1.000). Further characteristics are presented in Table 2.

**Table 2:** Characteristics of neonates with NAS

Neonatal abstinence syndrome	
NAS - n (%)	40 (50.6)
Age at diagnosis - median (IQR) [days]	2 (2)
ESC approach - n (%)*	
Poor breastfeeding / <30mL per feed	5 (12.5)
Sleep < 1 h	2 (5.0)
Difficult to console (> 10 min)	14 (35.0)
Highest FNAS - median (IQR) [points]	8.0 (2–23)
Pharmacologic treatment	
Methadone - n (%)	4 (9.8)
Duration of pharmacologic treatment - median (IQR) [days]	13 (21)

*The same neonate could have more than one of the ESC criteria; ESC, Eat, Sleep, Console; FNAS, Finnegan Neonatal Abstinence Score; IQR, interquartile range; NAS, neonatal abstinence syndrome

Consolability was the most affected criterion (*n* = 14; 35.0%), followed by feeding difficulties (*n* = 5; 12.5%). Sleep was affected to a lesser extent (*n* = 2; 5.0%). Exclusive breastfeeding was more common in neonates without NAS (53.8% *versus* 30.0%; *p* = 0.032), and rooming-in occurred in 50.0% of NAS cases *versus* 79.5% of neonates without NAS (*p* = 0.006).

Pharmacological treatment with methadone was employed in four cases (10.0% of neonates with NAS), with a median duration of 13 days. A detailed characterisation of these cases can be found in Table 3.

**Table 3:** Characteristics of neonates with NAS submitted to pharmacologic treatment

Newborn	1	2	3	4
**Birth year**	2010	2011	2014	2017
**Sex**	Male	Female	Female	Female
**Gestational age [weeks]**	38	35	38	38
**Birthweight [gram]**	2705	2675	2500	2625
**Milk**	Formula	Breast/Formula	Formula	Formula
**Total length of stay [days]**	28	13	30	16
**NICU admission** **(main cause)**	Yes (social)	Yes (jaundice)	Yes (social)	No (-)
**Length of stay at NICU [days]**	28	8	30	-
ESC approach*				
**Poor breastfeeding / <30mL per feed**	Yes	Yes	No	Yes
**Sleep < 1 h**	Yes	No	No	No
**Difficult to console (> 10 min)**	Yes	Yes	Yes	Yes
**Highest FNAS [points]**	20	17	23	19
**Age at NAS diagnosis [days]**	0	3	2	2
**Pharmacologic treatment**	Methadone	Methadone	Methadone	Methadone
**Duration of pharmacologic treatment [days]**	21	2	25	5

*After nonpharmacologic measures optimization; ESC, Eat, Sleep, Console; FNAS, Finnegan Neonatal Abstinence Score; NAS, neonatal abstinence syndrome; NICU, Neonatal Intensive Care Unit

Regarding neurodevelopmental outcomes, after a median follow-up time of 8 years, neuropshycomotor developmental delay was the most frequent diagnosis (13.9% of the overall sample). There were no differences between groups.

### Temporal trends

**Fig. 1:**
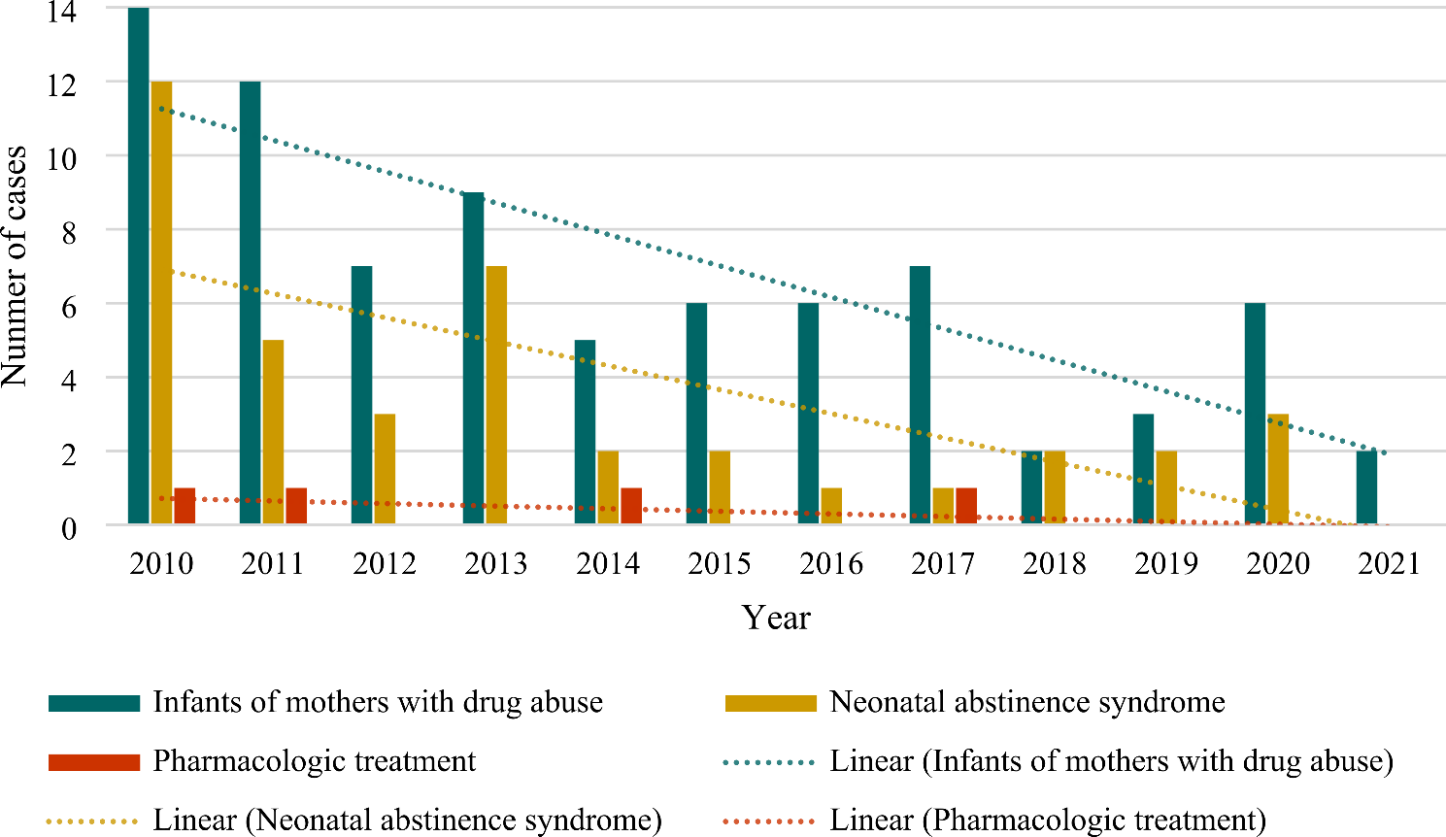
Evolution of the number of infants of mothers with drug abuse, cases of neonatal abstinence syndrome and infants needing pharmacologic treatment throughout the study period

**Fig. 2:**
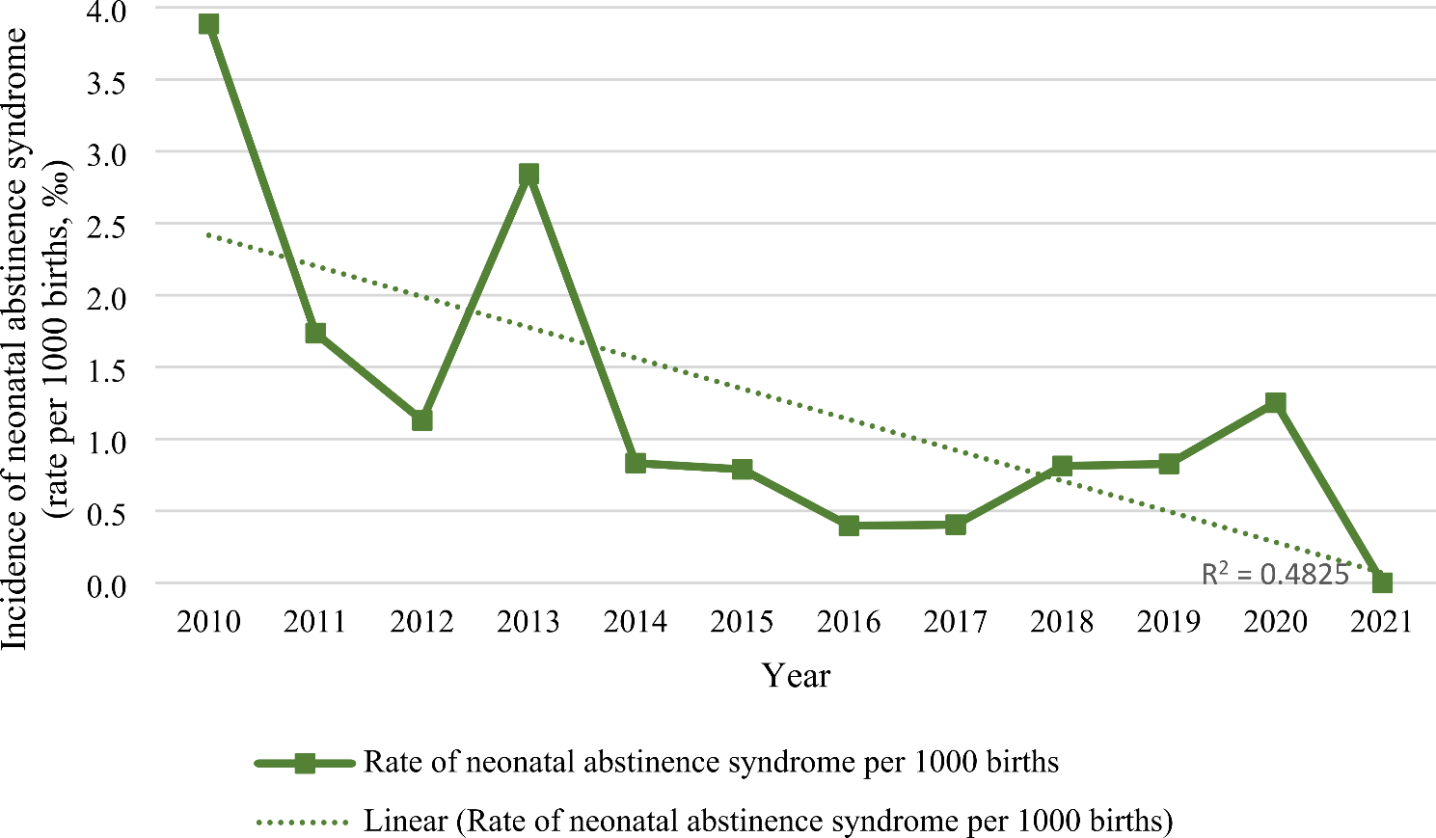
Rate of neonatal abstinence syndrome per 1000 births

Over the course of the study period, a reduction in the number of infants born to mothers with a history of drug abuse was observed, as well as a decline in the NAS cases and the need for pharmacological treatment (Fig. 1). The incidence rate of NAS significantly decreased from 3.9 per 1000 births in 2010 to 0.0 per 1000 births in 2021 (Kendall’s Tau − 0.529; p 0.025) (Fig. 2). The cumulative incidence of NAS during the study period was 1.2 cases per 1000 births.

There were no cases of NAS following discharge, and the mortality rate was 0%.

## Discussion

### Main findings

This study showed that the qualitative assessment of neonates at risk of NAS used at MBB was feasible and resulted in lower rates of NAS and need for opioid substitution therapy, confirming the high level of neonatal care provided.

Since the implementation of this system and the increased focus on the infant-mother dyad, the number of cases of NAS has decreased significantly. We hypothesise that this may be a consequence of the greater emphasis on non-pharmacological interventions. Furthermore, the incidence of NAS was lower than reported in the literature [9, 13, 15] and similar to a recent European study [36]. This may be due to the close monitoring of all cases by the multidisciplinary team of the EIU with early referral of pregnant women with drug abuse during the prenatal period. In addition, the high percentage of breastfeeding and rooming-in in this sample may also have contributed to this low prevalence. The beneficial effect of these factors in the management of neonates at risk of NAS has been previously reported [3, 4, 18, 37–40].

The use of pharmacological treatment was significantly lower compared with previous studies [1, 11, 16] and our own experience. In this centre, prior to the implementation of the current model of care, the use of the FNAS led to opioid substitution therapy in 81% of neonates with NAS [41]. With the introduction of the new newborn’s well-being qualitative assessment method in 2000 with a caregiver/infant-centred model of care, pharmacological treatment was significantly reduced to 10% without an increase in complications. This result is comparable to other authors using the ESC approach as an alternative tool to FNAS [9, 11, 21, 26, 42] and significantly better than older studies [1, 11, 16]. The greater focus on breastfeeding and non-pharmacological interventions in this population may have positively influenced these results, as several studies have shown their benefits in neonates at risk of NAS [3, 18, 38–40]. Furthermore, the qualitative method used at MBB correctly identified all infants who would have been selected using the extensively validated ESC approach.

The median length of stay for neonates with NAS was 8.5 days, significantly shorter than the one reported in most studies [17–20]. Grossman et al. [11], also reported a dramatic reduction from 22.5 to 5.9 days. As with the other improvements, this result may be due to intensive breastfeeding promotion and rooming-in.

Neonatal Abstinence Syndrome was more frequent in infants of mothers on heroin and/or multiple drugs, a finding previously reported in other studies [10, 43]. However, no other risk factors such as association with chronic medication were found. This may be explained by the small sample size. Methadone use was also higher among mothers of infants with NAS. The role of methadone as a risk factor for NAS remains controversial A meta-analysis showed no association between severity and maintenance dose [44], but more recent studies have demonstrated that higher doses may be linked to more severe NAS [45, 46]. We hypothesise that the higher percentage of methadone use in the NAS group is a result of more frequent heroin use. Unfortunately, as the dose of methadone at delivery was not available, we are unable to determine its potential impact on the study population.

Exposure to opioids and other illicit substances has been linked to impaired long-term neurodevelopmental outcomes, irrespective of the presence of NAS [47–51] In this study, no differences in long-term neurodevelopmental outcomes were observed. This finding may be justified by both the small sample size and the presence of disabilities irrespective of the development of NAS in the perinatal period, as prenatal exposure has been associated with these conditions [49–51].

Finally, an overall decrease in the number of cases of NAS and infants at risk of NAS during the study period was observed. This is in contrast to North America, where reported incidence rates of NAS have increased over the last decade [13, 14]. This discrepancy may be explained by the ongoing opioid crisis and differences in the public health system, less prescription of opioids, the limited availability of over-the-counter opioids and the established drug decriminalisation programme in Portugal.

### Strengths and limitations

To the best of our knowledge, this is the first European study to attempt to evaluate the potential impact of a non-pharmacological treatment approach on the development of NAS and the need for opioid substitution therapy. Most previous studies included only North American patients. Nevertheless, extrapolation of these results is difficult, because the type of drugs abused during the prenatal period and the pharmacological and non-pharmacological treatments differ significantly [52]. As a result, this study provides much needed evidence for an understudied subset of the population. Furthermore, it is one of the few available studies in Europe that reports on temporal trends of NAS.

This study also has some limitations. First, as a unicentric study in a tertiary Maternal and Child Hospital, the results must be interpreted with caution. However, as a referral centre for a region of almost two million people, we have extensive experience in caring for mothers with a history of substance misuse and children at risk of NAS. Secondly, as a retrospective study, some data may have been under-reported and it may be difficult to assess the non-pharmacological techniques used. Nevertheless, as each infant has an extensive and detailed in-hospital daily record, we believe that the data included in this study were precise and accurate. Thirdly, due to the qualitative nature of the assessment method used, it is difficult to retrospectively assess and quantify these parameters. However, as our institution undergoes frequent internal and external medical audits, we believe that the data included were as accurate and precise as possible for a study of this design. Finally, the last two years of the analysis included the COVID-19 pandemic, which may have resulted in fewer diagnoses. Nevertheless, the incidence of NAS clearly showed a decline over the last decade before the pandemic.

## Conclusion

In conclusion, a qualitative assessment of the infant based on the ability to feed, sleep and be consoled, with an emphasis on breastfeeding and optimisation of non-pharmacological treatment, can lead to a significant reduction in drug therapy with a potential ultimate benefit in clinical outcomes.

## Data Availability

All data generated or analysed during this study are included in this published article.

## Abbreviations

EIU: Early Intervention Unit
ESC: Eat, Sleep, Console
FNAS: Finnegan Neonatal Abstinence Score
NAS: Neonatal abstinence syndrome
NICU: Neonatal Intensive Care Unit
SD: Standard deviation
TORCH: toxoplasmosis, others (syphilis, hepatitis B), rubella, cytomegalovirus, and herpes simplex
